# A Response to the Legitimacy of Brain Death in Islam

**DOI:** 10.1007/s10943-016-0221-z

**Published:** 2016-03-24

**Authors:** Mohamed Y. Rady, Joseph L. Verheijde

**Affiliations:** Department of Critical Care, Mayo Clinic Hospital, 5777 East Mayo Boulevard, Phoenix, AZ 85054 USA; Department of Physical Medicine and Rehabilitation, Mayo Clinic, 13400 E Shea Blvd, Scottsdale, AZ 85259 USA

**Keywords:** Brain death, Fatwas, Organ donation, Islam, Law, Moral code, Neuroscience, Quran, Religion

## Abstract

Brain death is a novel construct of death for the procurement of transplantable organs. Many authoritative Islamic organizations and governments have endorsed brain death as true death for organ donation. Many commentators have reiterated the misconception that the Quranic text does not define death. We respond by clarifying: (1) the Quran does define death as biologic disintegration and clearly distinguishes it from the dying process, (2) brain death belongs scientifically within the spectrum of neurologic disorders of consciousness and should not be confused with death, and (3) religious and legal discord about brain death has grown in jurisdictions worldwide. We urge for public transparency and truthfulness about brain death and the accommodation and respect of religious objection to the determination of death by neurologic criteria.

## Introduction

Brain death is a novel Western construct of human death. Beecher and the Ad Hoc Committee of the Harvard Medical School to Examine the Definition of Brain Death (1968) introduced this particular concept of death in the USA. During the Committee’s deliberations, the input of theological, religious, and scientific considerations was minimized in order to expedite the approval of brain death as equivalent to human death (Rady and Verheijde 2013). Following the completion of the so-called Harvard criteria, the Uniform Law Commission first created the Uniform Brain Death Act in 1978, and later replaced this Act with the Uniform Determination of Death Act (UDDA) in 1980 (National Conference of Commissioners on Uniform State Laws 1981). The enactment of the UDDA was an essential step in advancing the US organ donation and transplantation practice because it legally permitted the procurement of vital organs from heart-beating donors (Rady and Verheijde 2013).

In spite of persistent scientific and religious controversies about brain death, the Resolution of the Council of Islamic Jurisprudence on Resuscitation Apparatus incorporated “brain death” as a definition of death in Islam (Rady and Verheijde 2013). This was followed by several worldwide Islamic organizations and governments also endorsing brain death as true death for the purpose of organ donation and transplantation (Miller 2015). The primary sources of the moral code in Islam are the Quran (God’s revelation to mankind) and the Sunnah (the actions and sayings of the Prophet) (Rady and Verheijde 2014). Miller (2015) has reiterated the claim generally made by Muslim scholars who have justified the endorsement of brain death that “there is neither a precise definition of death, nor a precise description of how to recognize the departure of the soul from the body in either the Qu’ran or Sunnah.” Such a claim is a necessary condition for establishing an opportunity to reinterpret the moral code for the purpose of integrating pragmatic, utilitarian medical objectives, and embracing controversial end-of-life practices in Muslim communities globally. A new discipline of “Islamic bioethics” was created for reconciling controversial secular medical practices, such as organ donation in brain death, with the moral code in Islam (Rady and Verheijde 2014). In response to Miller, we clarify the following pertinent points about brain death and Islam: (1) the Quran does define death and clearly distinguishes it from the dying process, (2) brain death belongs scientifically within the spectrum of neurologic disorders of consciousness and should not be confused with death itself, and (3) the religious and legal discord about brain death is growing in Western jurisdictions.

## The Quran Defines and Distinguishes Death from the Dying Process

The Quran emphasizes the singularity of the death phenomenon. Death is characterized by biologic disintegration. The Quran explicitly differentiates between the process of dying and death, the latter being the final outcome of that process (Rady and Verheijde 2014, 2015). The onset of disintegration begins after the completion of the dying process. In thermodynamic and biologic terms, disintegration is associated with an irreversible loss of thermodynamic entropy (internal energy generation) and the loss of homeostasis and cellular, tissue, organ, and whole body integration.

[D]ead biosystem discontinues the forcing of the energy into the biosystem’s compartments, which, in the living case, would transform into internal energy that could be used for the progression of the processes that permits the biosystem to maintain a quasi-stable entropic state (Nahle 2009).

Once cellular energy generation ceases, the biologic system begins disintegration and declines rapidly into thermodynamic equilibrium with the external environment (Gatenby and Frieden 2013). Death is biologically defined in the Quran by disintegration. This is emphasized in several Quranic verses:

And he puts forth for Us a parable, and forgets his own creation. He says: “Who will give life to these bones after they are rotten and have become dust?” (The Quran, n.d., Chapter 36 verse 78);

When we are dead and have become dust and bones, shall we (then) verily be resurrected? (16) (The Quran, n.d., Chapter 37 verse 16);

And they used to say: “When we die and become dust and bones, shall we then indeed be resurrected?” (The Quran, n.d., Chapter 56 verse 47).

In contrast, the process of dying is a gradual process over time during which the ceased vital functions are reversible (Rady and Verheijde 2014). The Quran also describes the dying process:

Say: “The angel of death, who is set over you, will take your souls, Then you shall be brought to your Lord.” (The Quran, n.d., Chapter 32 verse 11);

Then why do you not (intervene) when (the soul of a dying person) reaches the throat? (83) And you at the moment are looking on, (84) But We (i.e. Our angels who take the soul) are nearer to him than you, but you see not, (85) (The Quran, n.d., Chapter 56 verse 83–85);

Nay, when (the soul) reaches to the collar bone (i.e. up to the throat in its exit), (26) And it will be said: “Who can cure him (and save him from death)?” (27) And he (the dying person) will conclude that it was (the time) of parting (death); (28) (The Quran, n.d., Chapter 75 verse 26–28).

Miller (2015) refers to the departure of the soul from the physical body. We agree with Miller that the relationship between the onset of biologic disintegration and the departure of the soul is not known. First, the locus of the soul within the human body is unknown. Second, the exact time when the soul departs from the body is also unknown. The extraction of the soul confirms the helplessness of man to reverse biologic disintegration. The Quran offers a reminder that the knowledge of the locus and time of departure of the soul is beyond mankind’s comprehension:

And they ask you concerning the Ruh (the Spirit); Say: “The Ruh (the Spirit): is one of the things, the knowledge of which is only with my Lord. And of knowledge, you (mankind) have been given only a little (85) (The Quran, n.d., Chapter 17 verse 85).

## Neuroscience and Brain Death

We have summarized elsewhere the scientific flaws with the concept of brain death (Rady and Verheijde 2013). However, we will focus here on the most relevant aspects that directly contradict the Quran and violate the moral code.

First, it is claimed that brain death is human death because the individual has lost the capacity for consciousness (Shemie et al. 2014). However, the standard of the American Academy of Neurology in diagnosing brain death is the clinical determination of absence of external responsiveness, brainstem motor reflexes, and spontaneous respiratory drive (Wijdicks et al. 2010). There is no mention of a reliable neurologic examination or test that can be performed to validate or predict the irreversible absence of capacity for consciousness. Indeed, neuroscientific research has verified the failure of clinical examination to detect residual consciousness or awareness of the self and the external environment in patients with severely injured brains (Peterson et al. 2014). Brain death belongs to the broad spectrum of neurologic disorders of consciousness (Rady and Verheijde 2016). Although brain-dead patients suffer a devastating injury to the brain that is associated with severe disabilities, this should not be confused with being diagnosed as dead. The Quran describes death as “yaqīn” (Arabic word for absolute certainty): “And worship your Lord until there comes unto you the certainty (i.e., death) (99)” (The Quran, n.d., Chapter 15 verse 99). The moral code forbids speculation and conjecture in the process of death determination (Rady and Verheijde 2014). The process of brain death determination is at best (1) a descriptor of a specific set of severe neurologic disabilities and (2) speculative about the irreversible loss of capacity for consciousness or awareness.

Second, residual functions of the central nervous system, homeostasis, and somatic integration of the whole body persist in brain death. Most of the biologic functions that are characteristic of living humans also continue in brain-dead patients (Rady and Verheijde 2013). As mentioned earlier, the Quran defines death by disintegration. Neither the entire central nervous system nor the body undergoes disintegration in brain death. Therefore, endorsement of brain death as true death directly conflicts with the Quran. There are moral consequences from ignoring this conflict. If organs are procured from heart-beating donors, this will result in a harmful interference and causation of death of another human life (Rady and Verheijde 2014). The moral code, however, upholds the inviolability of life. The Quran also explicitly warns against such actions:

And do not kill yourselves (nor kill one another). Surely, Allah [God] is Most Merciful to you. (29) And whoever commits that through aggression and injustice, We shall cast him into the Fire, and that is easy for Allah. (30) (The Quran, n.d., Chapter 4 verse 29–30).

Therefore, the endorsement of worldwide Islamic organizations and governments of brain death as a substitute for the Quranic definition of death has negative sociocultural consequences in Muslim communities (Rady et al. 2009). Such an endorsement authorizes a secular and controversial end-of-life practice that contravenes the Quranic instruction on human dignity and sanctity of life. The Quran cautions those who are authorizing potentially harmful practices in Muslim communities:

They may bear their own burdens in full on the Day of Resurrection, and also of the burdens of those whom they misled without knowledge. Evil indeed is that which they shall bear! (The Quran, n.d., Chapter16 verse 25).

## Western Religious and Legal Discord on Brain Death

Miller (2015) mentioned that there is medical and legal discord regarding brain death in Muslim communities. Religious and legal challenges to brain death determination also exist in Western society. The President Council on Bioethics (2008) has outlined the scientific weakness underpinning the medical construct of brain death and its equivalency with death. In an attempt to salvage the definition of brain death and retain the utility of brain death in the US organ donation and transplantation practice, the President Council proposed a new philosophical rationale for determining death by neurologic criteria. Critics have argued that this philosophical rationale fails to validate the notion of equating brain death with human biologic death in the same way the old rationale did (Shewmon 2009, 2012). More importantly, abandoning the biologic definition of death and replacing it with one that is equally encumbered by philosophical and scientific shortcomings can have serious sociocultural consequences in Western societies. This risk is exacerbated when the general public is poorly educated about the facts and the potential moral implications of such change. The secular values assigned to the construct of brain death can transgress other dominant religious and cultural values in pluralistic societies. Indeed, this was illustrated in the case of Jahi McMath (Luce 2015). Jahi McMath was declared brain-dead in December 2013. Her parents objected based on religious grounds and transferred Jahi to a different medical facility for continued medical care (Johnson 2016). Jahi McMath has remained alive with mechanical ventilation and assisted nutrition. The case of Jahi McMath has alerted the US public about serious scientific flaws in the neurologic determination of death and an urgent need to accommodate and respect religious objection to brain death declaration (Johnson 2016). Some commentators have counter-argued that the continuation of medical treatment in Jahi McMath contravened the ethical obligation of health care professionals to “maintain patient’s dignity” (Lewis et al. 2016). Others have claimed that the denial of determination of death by neurologic criteria is “an affront to the dignity and respect for the dead” (Miller 2016). Miller and Lewis et al.’s interpretation of dignity appears paradoxical. Medical treatment is perceived as a violation of dignity if it is continued because of religious objection to brain death. On the other hand, the performance of invasive procedures for organ preservation (Kotloff et al. 2015), the surgical procurement of transplantable organs with no general anesthesia (Anderson et al. 2015), and the conduct of experimental human research with no consent (Glazier et al. 2015) on brain-dead persons are not considered violation of dignity.

There is also a discord between medical and legal definitions of brain death. The medical definition of brain death is seeking to maximize the opportunities for the procurement of transplantable human organs. Wijdicks et al. (2011) have re-affirmed that the 2010 American Academy of Neurology practice guidelines for determining brain death in adults did not fulfill the UDDA legal standard because “[t]e gold standard is not the UDDA but a neurologic examination and irreversible loss of all brainstem function.” The legal definition of brain death is seeking to ensure uniformity in determining death and, thus, protection from harm because of incorrect death determination (Rady and Verheijde 2016). The discord between medical and legal determination of brain death was brought to public attention in *Hailu vs Prime Healthcare* (Case No. 68531) (The Supreme Court of Nevada, 2015). The Supreme Court of Nevada has questioned whether the American Academy of Neurology practice guidelines, which are claimed to be the accepted medical standard for brain death determination, indeed adequately measure all functions of the brain, including the brainstem, as stipulated in the UDDA:

For legal and medical purposes, a person is dead if the person has sustained *anirreversible cessation of:* (a) Circulatory and respiratory functions; or (b) *All functions* of the person’s *entire brain, including his or her brain stem*…(emphasis added)*….* the UDDA sought to achieve greater uniformity in making such important and profound medical determinations….*Are the AAN [American Academy of Neurology] guidelines considered “accepted medical standards,” which adequately measure all functions of a person’s entire brain, including the brain stem?*…. Based on the foregoing and the record before us, we are not convinced that the AAN guidelines are considered the accepted medical standard that can be applied in a way to make Nevada’s Determination of Death Act uniform with states that have adopted it, as the UDDA requires (The Supreme Court of Nevada 2015) [italicized in the source document].

The Supreme Court of Nevada (2015) re-affirmed the legal importance of uniformity of brain death determination by the US medical community:

Brain death presents a mixed legal and medical question. Although “it is for [the] law to define the standard of death,” courts have deferred to the medical community to determine the applicable criteria for deciding whether brain death is present….Though courts defer to the medical community to determine the applicable criteria to measure brain functioning, it is the duty of the law to establish the applicable standard that said criteria must meet.

## Conclusions

The Quran differentiates between the process of dying and death itself. Death is defined as biologic disintegration. Irreversible cessation of homeostasis and cellular, tissue, and organ integration indicates biologic death. Brain death is a Western construct of death that has not been validated by neuroscience as equivalent to human death. Brain death is a neurologic state that belongs to the spectrum of disorders of consciousness and is associated with severe disabilities. The presence of severe neurologic disabilities should not be confused with death. The religious and legal discord with the medical construct of brain death is inevitable in pluralistic societies. Transparency and truthfulness about brain death is essential to avoid negative sociocultural consequences and to maintain trust in medicine. In addition to these moral recommendations, practical considerations should be given to accommodating and respecting religious objection to the neurologic determination of death.
